# Downregulation of Toll-Like Receptor Gene Expression Among Hepatitis B Virus-Positive Human Patients

**DOI:** 10.7759/cureus.90391

**Published:** 2025-08-18

**Authors:** Tamadhur H Hussein, Ameera Al-Yami, Nabil Mtiraoui, Jawhar Gharbi, Manel Ben M'hadheb

**Affiliations:** 1 Genomic, Biotechnology and Antiviral Strategies Research Unit (UR1ES30) DNA Analysis and Sequencing Unit of Common Services for Research (USCR-SAG), Institute of Biotechnology, University of Monastir, Monastir, TUN; 2 Department of Biological Sciences, College of Science, King Faisal University, Al-Ahsa, SAU; 3 Laboratory of Human Genome and Multifactorial Diseases (LR12ES07), Faculty of Pharmacy of Monastir, University of Monastir, Monastir, TUN

**Keywords:** gene expression, genotypes, hepatitis b virus, mrna, toll-like receptors

## Abstract

Background

Hepatitis B virus (HBV) chronically infects over several million people, often progressing to severe liver disease. Toll-like receptors (*TLRs*), notably *TLR9*, are critical for recognizing viral DNA and driving innate immune responses.

Objective

The objective of this study is to investigate the downregulation of *TLR* gene expression in HBV-infected patients, elucidating their role in innate immunity.

Methodology

A cohort of 434 HBV-positive patients was analyzed for *TLR* gene expression via real-time PCR.

Results

The HBV S genotype D (57.6%) was more prevalent than genotype C (42.4%) (p = 0.001). *TLR1*, *TLR2*, *TLR3*, *TLR4*, *TLR5*, *TLR6*, *TLR7*, *TLR8*, and *TLR9* were significantly downregulated in both genotypes as compared to controls, with genotype D showing greater downregulation of *TLR1*, *TLR5*, *TLR6*, and *TLR7* among HBV-positive patients.

Conclusion

Human* TLR* gene expression levels are downregulated due to HBV infection, with genotype D predominance suggesting differential immune regulation. These findings underscore the need for targeted HBV therapies informed by genotypic and immunological profiles.

## Introduction

Hepatitis B virus (HBV) infection is recognized as a major global public health concern, affecting over 250 million people and significantly increasing their risk of developing end-stage liver disease. In endemic regions, perinatal transmission, including intrauterine, intrapartum, and postpartum routes, is the predominant mode of HBV spread. Infection acquired early in life progresses to chronic infection in most cases (> 95%). Chronic HBV infection presents with a spectrum of clinical outcomes, ranging from asymptomatic carrier states to severe liver disease such as cirrhosis and hepatocellular carcinoma (HCC) [1]. The natural history of chronic HBV infection is commonly divided into three phases: immune tolerance, immune clearance, and inactive or residual, based on HBV DNA levels, hepatitis B e-antigen (HBeAg) status, and serum alanine aminotransferase (ALT) levels.

The discovery of mammalian Toll-like receptors (*TLRs*) has significantly advanced our understanding of viral recognition and innate immune defense. These receptors, part of the pattern recognition receptor (*PRR*) family, detect conserved microbial structures known as pathogen-associated molecular patterns (*PAMPs*) [2]. In humans, there are 10 members of this class of receptors (named *TLR1* to *TLR10*). (*TLR1-TLR10*) have been characterized. *TLR1, TLR2, TLR4, TLR5,* and *TLR6* are located on the cell surface, whereas *TLR3, TLR7, TLR8,* and *TLR9* are found within intracellular compartments such as the endoplasmic reticulum, endosomes, lysosomes, and endolysosomes [3].

*TLR9*, an essential component of both innate and adaptive immunity, is predominantly localized within endocytic vesicles of plasmacytoid dendritic cells (pDCs) and B lymphocytes. *TLR9* recognizes unmethylated *CpG* motifs typically found in bacterial and viral DNA and signals through a *MyD88*-dependent pathway involving TNF receptor-associated factor 6 (*TRAF6*). This leads to activation of nuclear factor-kappa B (*NF-κB*), cytokine production, and initiation of inflammatory responses. Upon ligand binding, *TLR9* recruits the *MyD88* adaptor protein to its Toll/interleukin-1 receptor (*TIR*) domain, triggering the formation of a cytoplasmic signaling complex composed of interleukin-1 receptor-associated kinases (*IRAK1* and *IRAK4*), *TRAF6*, and interferon regulatory factor 7 (*IRF7*). This signaling cascade culminates in the transcription of type I interferons (*IFNs*) [1].

While prior studies have demonstrated HBV-mediated suppression of *TLR* signaling, particularly via HBsAg and HBeAg, the influence of HBV genotypes on the extent and specificity of *TLR* downregulation remains poorly understood. Notably, the predominance of genotype D in regions such as Egypt and its potential to differentially modulate innate immune responses compared to other genotypes, such as genotype C, has not been systematically explored. This study addresses this gap by investigating the expression profiles of *TLR1-TLR9* in a large cohort of HBV-infected patients, with a focus on genotype-specific differences, to elucidate the mechanisms underlying immune evasion and inform targeted therapeutic strategies.

Therefore, the principal objective of this study is to investigate the downregulation of *TLR* gene expression in HBV-infected patients and elucidate its role in innate immunity. Understanding these mechanisms could inform the development of targeted immunotherapies to enhance antiviral responses and mitigate HBV-associated liver diseases.

## Materials and methods

Sample study and selection criteria

A total of 434 patients with chronic hepatitis B (CHB) were included in this study, recruited from the general outpatient department of Cairo Hospital, Egypt, from January 2022 and December 2023. Inclusion criteria included: (1) confirmed chronic HBV infection (HBsAg-positive for ≥6 months and detectable HBV DNA by PCR), (2) age ≥18 years, and (3) willingness to provide written informed consent. Exclusion criteria included: (1) co-infection with hepatitis C virus, hepatitis D virus, or HIV, (2) acute HBV infection, (3) history of immunosuppressive therapy, (4) autoimmune liver diseases, or (5) pregnancy. The control group consisted of 100 age- and sex-matched healthy volunteers with no history of liver disease, negative HBsAg, and negative IgG anti-HBV antibodies, recruited from the same hospital during routine health check-ups. Recruitment ensured geographic and demographic representation reflective of the Egyptian population. All patients provided written informed consent, based on clinical investigation, and were positive for HBV using a third-generation enzyme-linked immunosorbent assay (ELISA) kit.

The diagnosis of acute HBV infection was based on the recent onset of jaundice in individuals with no prior history of chronic liver disease and no alternative explanation for jaundice (such as drug-induced hepatitis, severe infections, cholestatic jaundice of pregnancy, eclampsia, hemolysis, elevated liver enzymes and low platelet count syndrome, or acute fatty liver of pregnancy). Additional diagnostic criteria included a serum bilirubin level of 2.0 mg/dL or higher and elevated transaminase levels, specifically, serum ALT levels exceeding 2.5 times the upper limit of normal (reference range: 3-26 IU/L) [4].

The sample size of 434 HBV-positive patients was determined based on a power calculation to detect a moderate effect size (Cohen’s d = 0.5) in TLR gene expression differences between HBV-infected patients and controls. Using a two-tailed student’s t-test with a significance level (α) of 0.05 and power (1-β) of 80%, a minimum sample size of 400 patients was estimated, accounting for potential dropouts and variability in gene expression data. The final cohort of 434 patients ensured sufficient statistical power to detect clinically meaningful differences.

Diagnosis of HBV by serological method

The diagnosis of HBV infection was based on the detection of HBsAg using an ELISA kit, as described by Arankalle et al. [5]. All patients were tested for HBsAg and IgM antibodies against the hepatitis B core antigen (IgM anti-HBc) using ELISA kits according to the manufacturer's instructions (Abbott, AXSYM System, Germany). Only patients who tested positive for HBsAg were included in this study. The control group consisted of apparently healthy individuals with no serological markers of acute or chronic viral hepatitis and who tested negative for IgG anti-HBV antibodies. Quantification of HBsAg was performed using the Architect QT assay (Abbott Diagnostics, Wiesbaden, Germany), following the manufacturer's instructions. In brief, HBsAg in the sample was captured by anti-HBs-coated microparticles, followed by the addition of an acridinium-labeled anti-HBs conjugate and chemiluminescent trigger solutions. The resulting chemiluminescence was measured in relative light units. The quantitative HBsAg (qHBsAg) calibration curve ranged from 0.05 to 250 IU/mL. Samples exceeding this range were diluted (1:20 or 1:250) using a provided diluent to ensure accurate quantification.

Additionally, HBeAg levels were determined using the Architect platform (Abbott Diagnostics). Quantitative HBeAg (qHBeAg) was assessed via an automated microparticle chemiluminescent immunoassay, as previously described. Although the commercial kit is not specifically designed as a quantitative assay, it provides a signal-to-cutoff ratio that is linear over a limited range. The reference standard for HBeAg, with a defined activity of 100 Paul Ehrlich (PE) IU/mL, was obtained from the Paul Ehrlich Institute (Langen, Germany). An in-house working standard was prepared from high-titer HBeAg-positive samples and calibrated against this reference. Dilutions were performed as needed, with a linear detection range of approximately 0.13 to 100 PE IU/mL. A standard curve was generated, and linear regression analysis was used to express the results in PE IU/mL.

Molecular detection of HBV DNA by PCR

DNA Isolation and Detection

Viral DNA was extracted from the serum samples of patients following the protocol described by Valentine et al. [6]. The HBV surface gene (S gene) was selected as the molecular target for PCR detection due to its high conservation across all HBV genotypes, ensuring robust and specific amplification. The S gene encodes the hepatitis B surface antigen (HBsAg), a key diagnostic marker, and its sequence variability facilitates accurate genotyping, which is critical for assessing genotype-specific effects on *TLR* gene expression. This choice enhances the sensitivity and specificity of HBV detection compared to other genomic regions, such as the core or polymerase genes, which may exhibit greater variability or lower diagnostic utility. Amplifications were carried out using an Eppendorf Mastercycler Nexus Gradient thermal cycler (Hamburg, Germany).

First PCR

The first-round PCR was conducted using 1 U of Taq DNA polymerase (5 U/μL) in a reaction mixture containing outer forward and reverse primers (4 pmol each, Table 1), 1X PCR Buffer I, 0.2 mM dNTP mix (10 mM), and 2 mM MgCl₂ (from a 5 mM stock). A total of 100 ng of extracted viral DNA was added to the mix, and the final reaction volume was adjusted to 20 μL. The thermal cycling conditions were as follows: initial denaturation at 94 °C for 8 minutes; 30 cycles of denaturation at 94 °C for 25 seconds, annealing at 56 °C for 30 seconds, and extension at 72 °C for 45 seconds; followed by a final extension at 72 °C for 10 minutes using a thermocycler 2770 (Applied Biosystems, Foster City, CA, US).

**Table 1:** List of primers, expected amplicon size, and annealing temperature

Oligo name	Sequence (5’-3’)	Annealing	Amplicon
		Temperature (°C)	Size (base pairs)
Outer primers	Forward: ATCGCTGGATGTGTCTGCGG	56	700
	Reverse: GGCAACGGGGTAAAGGTTCA		
Inner primers	Forward: TTAGGGTTTAAATGTATACCC	55.3	428
	Reverse: CATCTTCTTGTTGGTTCTTCTG		

PCR products were analyzed by electrophoresis on a 1.5% agarose gel prepared in 1X Tris-borate-EDTA (TBE) buffer and stained with ethidium bromide (250 ng/μL). Five µl of each PCR product was mixed with 1 μL of 6X loading dye and loaded into the gel. A 100 bp DNA ladder (Invitrogen) was also included as a molecular weight marker. Electrophoresis was run at 100 V for 30 minutes.

Second PCR

The second round of nested PCR was performed using 100 ng of the first-round PCR product as the DNA template. The reaction mix included inner forward and reverse primers (4 pmol each, Table 1), 1X PCR Buffer I (10X), 2 mM MgCl₂ (from a 25 mM stock), 0.2 mM dNTP mix (10 mM), and 1 U of Taq DNA polymerase (5 U/μL), for a total reaction volume of 20 μL. The thermal cycling profile began with an initial denaturation at 94 °C for 8 minutes, followed by 30 cycles of denaturation at 94 °C for 25 seconds, annealing at 55.3 °C for 1 minute, and extension at 72 °C for 45 seconds. A final extension was performed at 72 °C for 10 minutes.

Nested PCR products were analyzed by electrophoresis on a 1.5% agarose gel containing 0.5 μg/mL ethidium bromide (Sigma-Aldrich, St. Louis, Missouri, US). Visualization was carried out using the Gel Doc 2000 imaging system (Bio-Rad, Hercules, California, US).

Genotyping of the HBV S Gene

HBV DNA was extracted from serum samples of both antiviral treatment responders and non-responders using a commercial viral DNA extraction kit (Favorgen Biotech Corporation, Pingtung, Taiwan), following the manufacturer’s protocol. The extracted DNA was stored at −20 °C until further use. Both responder and non-responder DNA samples were subjected to amplification of the S gene.

Amplification used primers targeting the S gene [7]: 5′GAACAAGAGCTACAGCATGGG3′ (forward primer) and 5′CTTTTGTCTTTGGGTATACAT3′ (reverse primer). The PCR reaction mixture had a final volume of 50 μL, comprising 3 μL of extracted DNA, 5 μL of forward primer (10 pmol/μL), 5 μL of reverse primer (10 pmol/μL), 25 μL of 2X Taq polymerase master mix, and 12 μL of nuclease-free water. Amplification was performed using a T100™ Thermal Cycler (Bio-Rad) under the following conditions: initial denaturation at 95 °C for 5 minutes; 30 cycles of denaturation at 95 °C for 30 seconds, annealing at 57 °C for 30 seconds, and extension at 72 °C for 30 seconds; followed by a final extension step at 72 °C for 10 minutes. PCR products were resolved on a 2% agarose gel pre-stained with ethidium bromide and visualized using a gel documentation system (Uvitec Ltd., Cambridge, UK).

Phylogenetic Analysis

The amplified S gene fragments were excised from the gel and purified using a gel purification kit (Thermo-Fisher Scientific, Waltham, MA, USA), according to the manufacturer's guidelines. Sequencing of purified PCR products was performed at Macrogen, South Korea, using the BigDye Terminator sequencing method (Applied Biosystems), facilitated by a private biotechnology service provider (Worldwide Scientific, Islamabad, Pakistan).

To assess genetic relationships, a phylogenetic tree was constructed using the obtained S gene nucleotide sequences from both responder and non-responder samples. Reference HBV surface gene sequences representing various global regions were retrieved from the NCBI database. Sequence alignment and phylogenetic tree construction were conducted using MEGA version 4 software.

*TLR* expression quantification by qRT-PCR

Total RNA Isolation

Total RNA was isolated from whole blood cells lysed with Trizol using the phenol-chloroform method, as described below [8]. Briefly, 200 µL of whole blood was mixed with 750 µL of Trizol reagent and 20 µL of 5N acetic acid in a microcentrifuge tube. The mixture was gently mixed and incubated at room temperature for five minutes. Then, 200 µL of chloroform was added, followed by brief vortexing and incubation at room temperature for another five minutes. The tubes were then centrifuged at 12,000 rpm for 15 minutes at 4 °C. The upper aqueous phase was transferred to a fresh microcentrifuge tube, and 250 µL of isopropanol was added. The tubes were incubated at -20 °C for 30 minutes and then centrifuged at 12,000 rpm for 10 minutes at 4 °C. After discarding the supernatant, the pellet was washed with 500 µL of 75% ethanol and centrifuged at 10,000 rpm at 4 °C for 10 minutes. The supernatant was discarded, and the pellet was air-dried. Finally, the pellet was resuspended in 20 µL of nuclease-free water and immediately used for cDNA synthesis following RNA quantification.

Complementary DNA (cDNA) Preparation

The cDNA synthesis was conducted using the high-capacity cDNA RT kit (Applied Biosystems). For the synthesis, 500-600 ng of extracted RNA was combined with 1X RT buffer (prepared from 10X stock), 0.4 mM dNTPs (from 100 mM stock), 0.02X RT random primer (from 10X stock), 50 U of reverse transcriptase (50 U/µL), and 20 U of RNase inhibitor (40 U/µL), with the final volume adjusted to 20 µL using nuclease-free water. The reaction mixture was briefly centrifuged and then subjected to thermal cycling.

The cDNA synthesis procedure consists of sequential temperature and duration steps. In the first step, the reaction is incubated at 25 °C for 10 minutes. In the second step, the temperature is elevated to 37 °C and held for 120 minutes. The third step involves heating to 85 °C for 4 minutes. In the final step, the reaction is cooled to 4 °C and maintained indefinitely.

The resulting cDNA was stored at -20°C for subsequent experiments.

mRNA Quantification of TLRs

Cells were collected, and total RNA was extracted using TRIzol reagent (Invitrogen, CA, USA) according to the manufacturer's instructions. A total of 2 µg of RNA was reverse-transcribed using M-MLV Reverse Transcriptase (Promega, Madison, WI, US) to generate cDNA samples. These cDNA samples were then used as templates for real-time PCR reactions.

Real-time PCR reactions were performed using the SYBR Green PCR Master Mix kit (Applied Biosystems). Each sample was analyzed in duplicate, and amplification was carried out using the 7300 Real-Time PCR System (Applied Biosystems). Each reaction mixture consisted of cDNA, a pair of primers (Table 2), and the PCR Master Mix in a final volume of 25 µL. A negative control, which contained all PCR components except cDNA, was included in each reaction. Additionally, a reverse transcription control was included, in which a sample lacking the SuperScript III RT enzyme was used as a template in real-time PCR.

**Table 2:** Primer sequences used in mRNA expressions of TLRs by real-time PCR

Gene	Size (bp)	Primer sens	Primer sequences (5’ to 3’)	Annealing temperature (°C)
TLR1	512	Forward	CCTCAAGCACTTGGACCTGTC	50
		Reverse	GACCCTGTAGCTTCACGTTTG	
TLR2	465	Forward	AGAGTTTGATGACTGTACCC	50
		Reverse	CTTTTCTGGCCACTGACAAG	
TLR3	255	Forward	CTGGAAGAAAGGGACTTTGA	55
		Reverse	CCTCTTCGCAAACAGAGTGC	
TLR4	206	Forward	AGCCGCTGGTGTATCTTTGA	48
		Reverse	CTGAGTCGTCTCCAGAAGAT	
TLR5	549	Forward	AGAATCCCAGCTTAGAACAG	
		Reverse	AGGAACAGAGTCAGAGTGAC	50
TLR6	110	Forward	GATTTCTTCCAGAGCTGCCAG	
		Reverse	CGTAATGGCACCACTCACTC	50
TLR7	262	Forward	GGAAAACCTTTCCCAGAGCA	
		Reverse	CTGCCAGAAGTATGGGTGAG	55
TLR8	268	Forward	GACAACCTCATGCAGAGCAT	
		Reverse	TCAGAGTTTGCCAAAACAAG	54
TLR9	597	Forward	CTGGCTGTTCCTGAAGTCTG	
		Reverse	GCGGTCAGATTGGCCAGGTC	59
GAPDH	250	Forward	CCACTGGCGTCTTCA	
		Reverse	ATGAGTCCTTCCACGATACC	52

After an initial six-minute denaturation step at 94 °C, 35 amplification cycles were performed. Each cycle consisted of three steps: denaturation (1 minute at 94 °C), hybridization (1 minute at the appropriate temperature), and elongation (1 minute at 72 °C). A final elongation step was performed for 7 minutes at 72 °C. At the end of each reaction, a dissociation curve was plotted to confirm that each reaction produced a single amplicon, as indicated by a single dissociation peak [9].

Data analysis

Statistical analyses were performed using R Project for Statistical Computing (version 4.2.2; R Core Team (2021). R: A language and environment for statistical computing. R Foundation for Statistical Computing, Vienna, Austria). Pearson’s chi-squared test was used to assess inter-group significance for categorical variables (e.g., sex, smoking status, and HBV genotypes). Student’s t-test was employed to compare means of continuous variables (e.g., *TLR* gene expression levels) between HBV-infected patients and controls. To account for multiple comparisons in the analysis of nine *TLR *genes, p-values were adjusted using the Benjamini-Hochberg correction to control the family-wise error rate, with a significance threshold of p < 0.05. Relative *TLR* gene expression was quantified using the 2^(-∆∆CT) method, normalized to *GAPDH*, and expressed as fold change relative to controls. All tests were two-tailed, and results with adjusted p-values < 0.05 were considered statistically significant [10].

To account for the significant sex imbalance in the cohort (79.9% male, p = 1.17e-35), a sex-stratified analysis of *TLR* gene expression was performed using the student’s t-test to compare fold change values between male and female HBV-infected patients, with Bonferroni correction applied for multiple comparisons.

The housekeeping gene *GAPDH *was selected as the reference gene for normalization due to its stable expression across HBV-infected and control samples. To validate *GAPDH *stability, cycle threshold (Ct) values were analyzed in a subset of 50 HBV-infected and 50 control samples. The mean Ct values for *GAPDH *were 20.3 ± 0.8 in HBV-infected samples and 20.1 ± 0.7 in controls, with no significant difference (p = 0.62, student’s t-test), confirming its suitability as a stable reference gene for qRT-PCR normalization in this study.

## Results

Analysis of demographic and clinical characteristics

The baseline characteristics of the 434 patients enrolled in the study are summarized in Table 3: 87 patients (20.04%) were female, while the majority, 347 patients (79.9%), were male (P-value = 1.17e-35). According to our results, 207 patients (47.69%) were non-smokers, whereas 227 patients (52.30%) reported being smokers (P-value = 0.337). Regarding diabetes history, 94 patients (21.65%) had no prior diagnosis, while 340 patients (78.34%) had a known history of diabetes (P-value = 4.22e-32). Compared to non-obese patients, obese patients had significantly higher mean values of BMI (P-value = 2.25e-28).

**Table 3:** Demographic data of the 434 positive samples of hepatitis virus B (HBV) † Pearson square nominal P-value; ‡ Benjamini-Hochberg adjusted P-value; FDR: false discovery rate

Parameters		Frequency (n)	Percent (%)	P-value^†^	FDR P-value^‡^
Sex	Male	347	79.9	<0.001	<0.001
	Female	87	20.04		
Smoking	Nonsmoker	207	47.69	0.337	0.337
	Smokers	227	52.30		
Diabetes mellitus	No	340	78.34	<0.001	<0.001
	Yes	94	21.65		
Body mass index (kg.m^-2^)	< 25	102	23.50	<0.001	<0.001
	> 25	332	76.49		
Antiviral treatment	Lamivudine	124	28.57	0.012	0.024
	Entecavir	173	39.86		
	Tenofovir	137	31.56		
HBV DNA (IU/mL)	< 2000	4	0.92	<0.001	<0.001
	2000 – 20000	354	81.56		
	> 20000	76	17.51		

With respect to HBV treatment, 124 patients (28.57%) were receiving Lamivudine, 173 patients (39.86%) were on Entecavir, and 137 patients (31.56%) were treated with Tenofovir (P-value = 0.012). When looking at HBV DNA levels (IU/mL), 354 patients (81.56%) had levels ranging between 2000 and 20,000 IU/mL, while 76 patients (17.51%) had levels exceeding 20,000 IU/mL (P-value = 3.18e-103).

Genotyping and phylogenetic study of the HBV S gene among patients

Detection of HBV DNA by PCR

**Figure 1:**
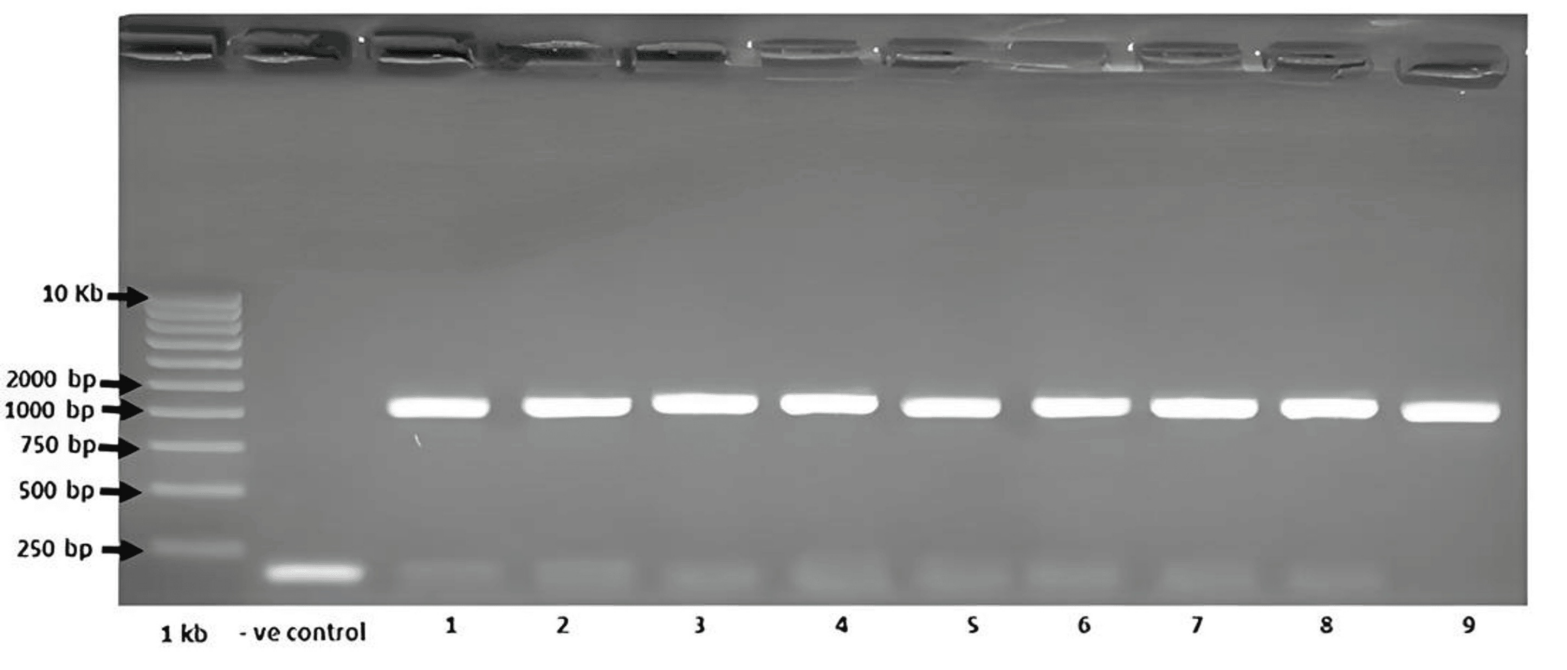
Gel electrophoresis of the PCR amplicon obtained from HBsAg First lane M: 1Kbp DNA ladder, second lane: negative control ( -ve control), lanes 1 to 9 represent amplification results for HBsAg

To confirm the presence of the S gene DNA in the samples, the PCR method was employed. A detailed evaluation of the gel images was conducted, and the results for all samples studied are shown in Figure 1. The data were compared to the negative control and analyzed accordingly. The results confirmed the presence of S gene DNA in the samples. Further confirmation was obtained by sequencing the amplified products. Sequence analysis indicated that the amplified product represented a full-length, wild-type HBV genome, suggesting that the sample was heavily contaminated with the virus.

Genotyping and phylogenetic study of HBV S gene strains

Two predominant genotypes of the S gene were identified across all samples: genotype C and genotype D. Genotype D was the most common, found in 250 of the samples (57.58%), while genotype C was present in 184 of the samples (42.42%). These findings highlight a distinct difference between the two genotypes (P-value = 0.001).

**Figure 2:**
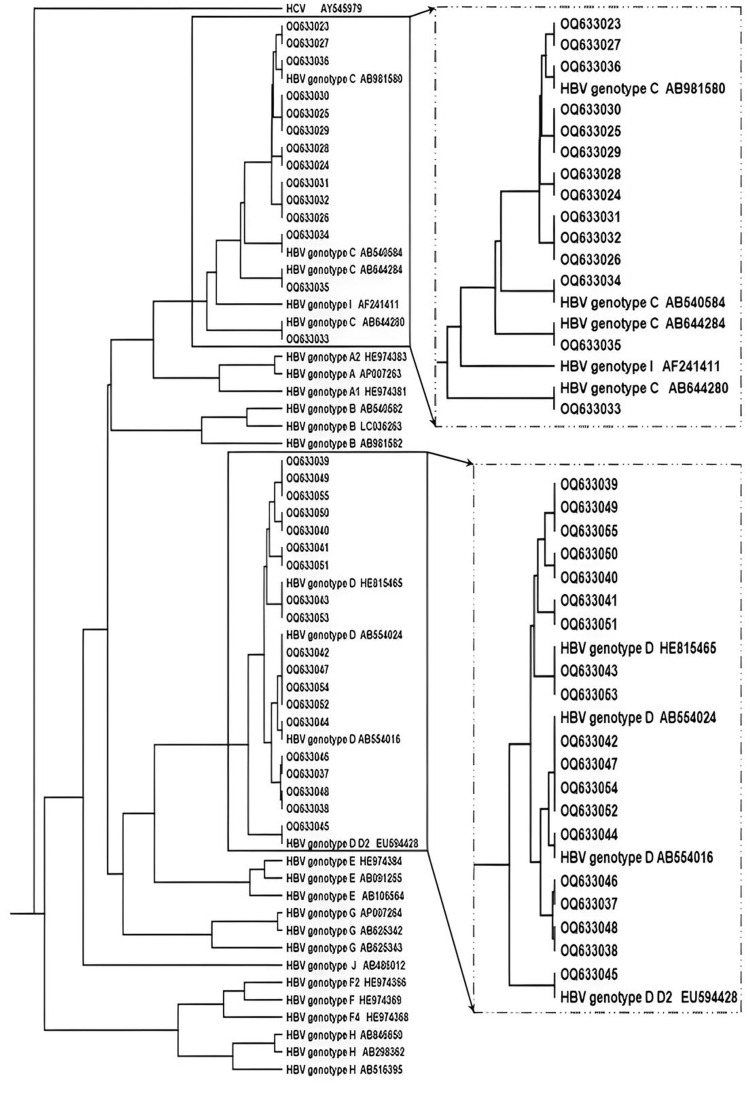
Phylogenetic tree constructed for the HBV S gene using 33 isolates with HBV genotypes (A-J) retrieved from the GenBank database Isolates from the current study are presented by registered accession number at the GenBank database. The tree was constructed using the maximum likelihood method (MEGA 4.0 software).

The 33 S gene sequences generated in this study were submitted to GenBank under accession numbers #OQ633023 to #OQ633055 and are publicly accessible at https://www.ncbi.nlm.nih.gov/genbank/ and https://www.ncbi.nlm.nih.gov/genbank/. Using these sequences, a phylogenetic tree was generated, and the alignments were performed in ClustalX2 against standard HBV sequences for genotypes D and C using MEGA 4.0 software (Figure 2). The study compared nucleotide-level multiple sequence alignments of representative HBV isolates with sequences from various global regions to assess relatedness. As indicated by the findings, the genotype most closely resembles those found in Egyptians and other African populations. This suggests that the HBV genotype in Egypt may be significantly older than previously believed and could have spread to other regions through population movements. This spread likely contributed to the global distribution of this genotype.

Expression of *TLR* genes among infected human patients

**Figure 3:**
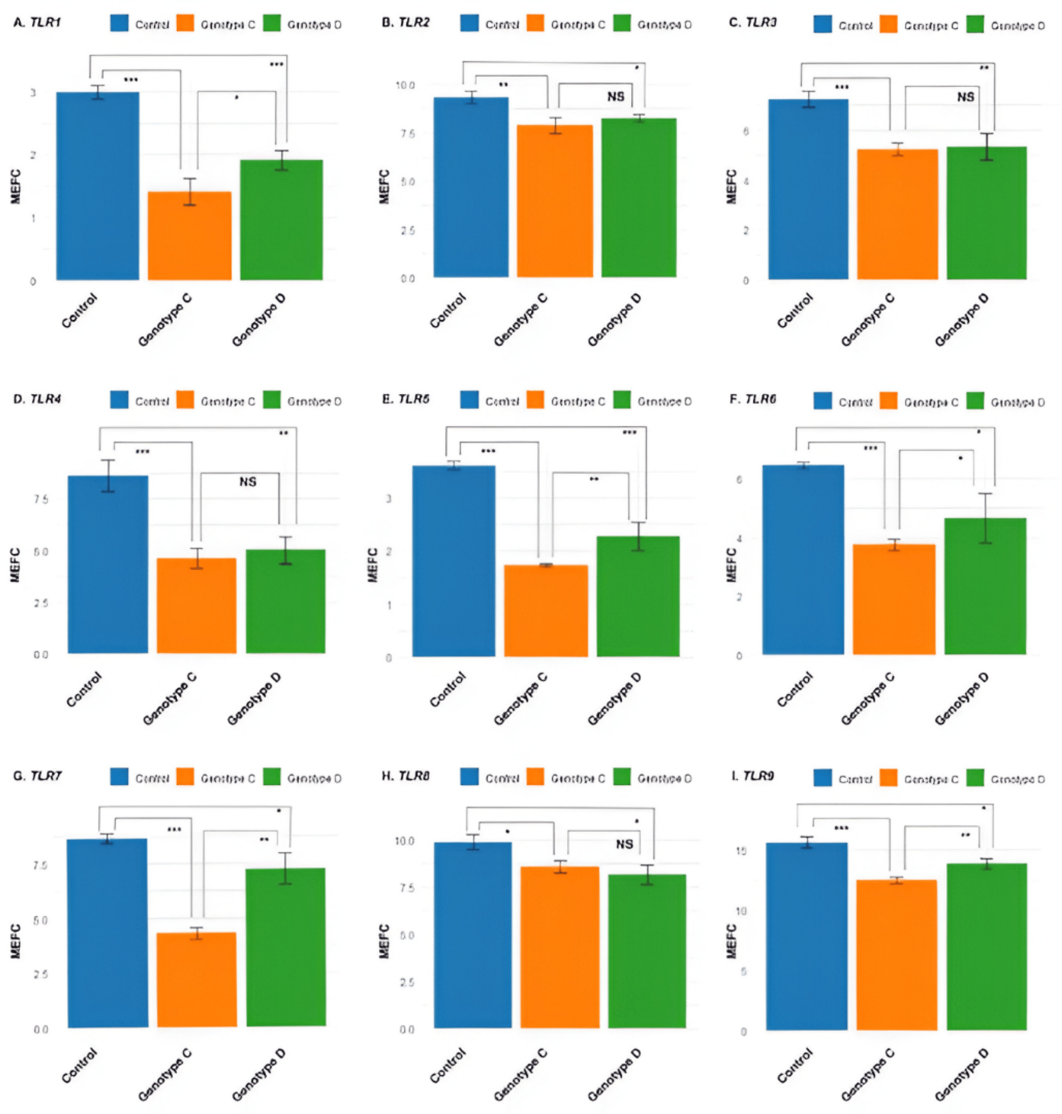
Mean expression fold change of nine TLR genes among hepatitis B virus-positive human patients MEFC: mean expression fold change, NS: non-significant, * : P-value < 0.05, ** : P-value < 0.01, *** : P-value < 0.001

Real-time PCR, using SYBR green dye and gene-specific primers, was conducted to evaluate the expression profiles of *TLR* genes. Expression levels in both treated and control cell line samples were quantified through fold change analysis, based on the Ct values of the target and housekeeping genes (Figure 3). Fold change comparisons were then performed for each gene across various categories, followed by comparisons between the categories themselves. *TLR1* expression was significantly downregulated in both genotypes compared to the control. However, genotype D exhibited a more pronounced downregulation than genotype C, suggesting that genetic variation may influence *TLR1* expression. In contrast, *TLR2* expression was also significantly reduced in both genotypes relative to the control, but no difference was observed between genotypes D and C, indicating the involvement of a shared regulatory factor.

Similarly, *TLR3* and *TLR4* showed significantly reduced expression in both genotypes compared to the control, with no significant differences between genotypes, again suggesting a common mechanism underlying their downregulation. For *TLR5, TLR6,* and *TLR7*, both genotypes demonstrated substantial downregulation compared to the control, but genotype D exhibited significantly lower expression levels than genotype C. This points to genotype-specific genetic differences influencing the regulation of these genes.

*TLR8* and *TLR9* were also significantly downregulated in both genotypes compared to the control. However, their expression levels were similar in genotypes D and C, implying that a common factor, possibly a master regulator, may control their expression in both genotypes. Further investigation is needed to identify this regulator and clarify the mechanisms behind the observed gene expression patterns.

## Discussion

The immune response to HBV infection is complex, involving both innate and adaptive immunity. Effective control requires generating sufficient HBV-specific T and B cells. Recently, innate immunity has been recognized for its role in clearing HBV and suppressing the virus during chronic infection. Moreover, antiviral drugs may stimulate innate immunity, further enhancing viral clearance [11].

As one of the first lines of defense against viral infections, *TLRs* play a crucial role by initiating intracellular signaling pathways that activate antiviral mediators such as interferons (IFNs) and other cytokines [12]. These mediators then induce the expression of immune-related molecules, facilitating the clearance of viruses from infected cells. Furthermore, *TLRs* are capable of recognizing viral components, which trigger the production of chemokines and cytokines, leading to the recruitment of immune cells to the site of infection [13].

Studies have shown that the activation of *TLR*-mediated signaling pathways can reduce HBV replication in both in vitro and in vivo settings. However, HBV has evolved mechanisms to counteract *TLR* responses such as suppressing *TLR* expression and blocking downstream signaling pathways [14]. When treated with antiviral agents, patients with chronic HBV infections experience upregulation of *TLR* receptors, restoring the innate antiviral function [15]. Thus, activating *TLRs* may represent a promising immunotherapeutic strategy to treat chronic HBV infection in conjunction with antiviral therapies, potentially improving efficacy. *TLR*-based immunotherapy could also reduce the risk of HBV-induced liver diseases, such as cirrhosis and hepatocellular carcinoma [16].

This study aimed to evaluate the impact of HBV isolates on *TLR* transcription and gene expression across different genotypes in selected samples and to determine the statistical significance of these effects. The researchers sought to explore how HBV isolates regulate *TLR *expression in various genetic backgrounds and to assess the relevance of the observed effects.

The significant sex imbalance in our cohort (79.9% male) reflects the higher prevalence of chronic HBV infection among males in Egypt, consistent with prior studies reporting elevated HBV exposure in men due to occupational and behavioral risk factors [17]. To assess potential confounding effects of sex on *TLR* gene expression, a sex-stratified analysis was conducted, revealing no significant differences in *TLR* downregulation patterns between males and females (p > 0.05 for all *TLR* genes). These findings suggest that the observed *TLR* downregulation is primarily driven by HBV infection rather than sex-specific factors, though further studies with balanced cohorts are needed to confirm this. Further research is needed to explore why this difference exists and to identify strategies to reduce HBV prevalence in males. These findings align with those of Spearman et al., who estimated that HBV prevalence among chronically infected individuals worldwide is higher in men, with related diseases and carcinomas also being more prevalent in males [17]. In 2005, the prevalence of chronic HBV infection was 3.5% in men and 3.9% in women. The increasing prevalence of HBV is particularly concerning, as it is the leading cause of death in individuals with chronic HBV infection, emphasizing the need for improved public health measures.

Our study also found detectable levels of HBV viremia in all analyzed cases, consistent with previous research. When comparing chronic hepatitis B (CHB) cases to total viremia, a difference was noted. While total viremia levels were high, CHB levels were lower, suggesting that total viremia may be a more useful predictor of HBV infection progression than CHB itself [18]. However, further research is required to confirm these findings and understand the role of CHB in HBV infection. Additionally, other markers, such as HBsAg, should be considered when predicting the progression of HBV infection.

Clinical associations with specific HBV genotypes are becoming more evident, leading to a growing demand for genotyping of patient strains. The HBV genome is divided into 8 genotypes based on an intergroup divergence of more than 8%, with different genotypes being more prevalent in different regions. Understanding the global diversity of HBV genotypes is crucial for the development of effective vaccines and treatments. Knowledge of genotype prevalence can also help tailor interventions and treatments to specific populations [19].

Our results indicated that genotype D was the predominant HBV genotype in the studied subjects, consistent with findings from other studies conducted in Egypt. Saudy et al. reported that genotype D was the most prevalent in Egypt, based on DNA sequencing of isolates from 100 serum samples [20]. However, they also noted the limitations of sequence analysis, as it can only detect the majority strain. For a more accurate genotype profile, additional methods like restriction fragment length polymorphism should be used along with sequencing. This would allow for a more thorough assessment of the genotypes present.

Similarly, studies by Zekri et al. also confirmed that genotype D is the predominant HBV genotype in the Mediterranean region and the Middle East [21]. Genotype D prevalence is particularly high in Egypt, as shown by studies in Turkey and other regions [22,23]. This variability in HBV genotype distribution highlights the need for region-specific studies to better understand the disease's epidemiology.

In this study, we confirmed the genotype of the samples as HBV genotype D through phylogenetic analysis, which showed slight differences compared to previously registered isolates in GenBank. These results were consistent with a similar study by Tatematsu et al., which used phylogenetic analysis to assess the genetic sequences of HBV isolates [24].

HBV is considered a "stealth" virus, as it does not activate innate immune system genes during acute infection, unlike other hepatotropic viruses such as HCV. This allows HBV to evade detection during viral entry and replication, increasing the likelihood of chronic infection. This evasion mechanism contributes to HBV's high prevalence, as it can persist in the host for extended periods [25].

Studies have shown that the immune response to HBV infection activates the *TLR* signaling pathway, which helps suppress HBV replication. Modulating this pathway could offer a potential treatment strategy for chronic HBV infections. Furthermore, research has indicated that HBV can suppress *TLR*-mediated innate immunity in hepatocytes and nonparenchymal liver cells, potentially by proteins such as HBsAg and HBeAg [26]. Understanding how HBV manipulates *TLR* responses is key to developing new therapeutic strategies.

Moreover, studies have demonstrated that HBsAg inhibits *TLR2* signaling, which is critical for activating cytokine production and immune responses [27]. This suggests that HBsAg may serve as an important regulator of the immune response in chronic HBV infection, highlighting its potential as a therapeutic target.

Our study also observed significant reductions in *TLR2* expression in HBsAg-positive patients during the protracted stages of infection, indicating that persistent infection may play a stronger role in suppressing TLR expression as the disease progresses. Monitoring *TLR* expression in HBsAg-positive patients could serve as a useful indicator of infection severity and could inform therapeutic interventions.

Finally, our study found that *TLR3 *expression was downregulated in HBV-infected patients, a result consistent with previous studies that identified *TLR3* as a potential target for treating HBV-mediated CHB [28]. Similarly, the downregulation of *TLR7* and *TLR9 *expression in HBV-infected individuals suggests that these receptors play a crucial role in the antiviral response. Targeting *TLR9*-mediated signaling could be a promising strategy for developing new therapies for CHB [29]. Further studies are needed to better understand the role of *TLRs* in HBV infection and to explore potential therapeutic strategies targeting these receptors.

This study has several notable strengths. First, the large cohort of 434 HBV-positive patients provides robust statistical power to detect differences in *TLR* gene expression. Second, the detailed methodological descriptions, including PCR protocols, primer sequences, and statistical analyses, ensure reproducibility and transparency. Third, the genotyping of the* *S gene and phylogenetic analysis offer valuable insights into the predominance of genotype D in Egypt and its potential impact on immune responses. Finally, the comprehensive analysis of all nine *TLR* genes (*TLR1-TLR9*) provides a holistic view of innate immune modulation in HBV infection, contributing to the understanding of genotype-specific immune evasion mechanisms.

We have to notice that this study has several limitations that should be considered when interpreting the results. First, the observational and cross-sectional design precludes establishing causation between HBV infection and *TLR* downregulation, limiting our findings to associations. Second, the study was conducted at a single hospital in Cairo, Egypt, which may limit the generalizability of the results to other populations or regions with different HBV genotype distributions. Third, the absence of functional experiments restricts our ability to elucidate the mechanistic impact of *TLR* downregulation on immune responses. Additionally, potential confounding factors, such as the effects of antiviral treatments (e.g., Lamivudine, Entecavir, Tenofovir) on *TLR* expression, were not fully explored. Finally, while the control group was age- and sex-matched, detailed clinical characteristics were not comprehensively reported, which could affect the robustness of comparisons. Future studies addressing these limitations are warranted to validate and extend our findings.

## Conclusions

HBV remains a significant global health challenge due to its high chronicity and association with severe liver diseases. This study demonstrates a significant association between HBV infection, particularly genotype D, and the downregulation of *TLR1, TLR2, TLR3, TLR4, TLR5, TLR6, TLR7, TLR8,* and *TLR9* gene expression in infected patients compared to controls. Notably, genotype D exhibited more pronounced downregulation of *TLR1, TLR5, TLR6,* and *TLR7*, suggesting potential genotype-specific differences in immune modulation. These findings, based solely on mRNA expression data, indicate an association with HBV’s possible immune evasion strategies, potentially involving HBsAg and HBeAg, though functional studies are needed to confirm these mechanisms. However, the cross-sectional design and lack of protein-level or functional assays preclude causal inferences about immune evasion mechanisms. The predominance of genotype D in our cohort underscores the importance of region-specific research. While these results suggest that *TLR*-based immunotherapy could be a promising avenue for enhancing antiviral responses, such implications are preliminary and hypothesis-generating. Longitudinal and mechanistic studies are essential to validate these associations and explore their therapeutic potential.

Based on the findings of this study, we propose the following recommendations for future research: a) Longitudinal studies should be done to track *TLR* gene expression over time in HBV-infected patients to better understand the temporal dynamics of immune modulation and its association with disease progression; b) It is very important for further studies to perform functional experiments, such as in vitro or in vivo studies, to elucidate the mechanisms by which HBV genotypes, particularly genotype D, downregulate TLR expression and impact innate immune responses; c) To expand the study, diverse populations should be included from different geographic regions to assess the generalizability of *TLR* downregulation patterns and their association with HBV genotypes. Confounding factor analysis is very important in the investigation of the impact of antiviral treatments and other clinical factors (e.g., diabetes, metabolic syndrome, obesity) on *TLR* expression to account for potential confounders. It will be interesting to explore the potential of *TLR* agonists as adjunctive therapies in combination with existing antiviral treatments to enhance immune responses in chronic HBV patients.
